# Study of Al-Si Alloy Oxygen Saturation on Its Microstructure and Mechanical Properties

**DOI:** 10.3390/ma10070786

**Published:** 2017-07-11

**Authors:** Arkady Finkelstein, Arseny Schaefer, Olga Chikova, Konstantin Borodianskiy

**Affiliations:** 1 Department of Foundry Engineering and Strengthening Technologies, the Ural Federal University, Yekaterinburg 620002, Russia; avinkel@mail.ru (A.F.); arsenii_shefer@mail.ru (A.S.); 2 Department of Physics, the Ural Federal University, Yekaterinburg 620002, Russia; chik63@mail.ru; 3 Zimin Advanced Materials Laboratory, Department of Chemical Engineering, Biotechnology and Materials, Ariel University, Ariel 40700, Israel

**Keywords:** aluminum alloy, modification, aluminum oxides, oxygen blowing

## Abstract

One of the main goals of modern materials research is obtaining different microstructures and studying their influence on the mechanical properties of metals; aluminum alloys are particularly of interest due to their advanced performance. Traditionally, their required properties are obtained by alloying process, modification, or physical influence during solidification. The present work describes a saturation of the overheated AlSi_7_Fe_1_ casting alloy by oxides using oxygen blowing approach in overheated alloy. Changes in metals’ microstructural and mechanical properties are also described in the work. An Al_10_SiFe intermetallic complex compound was obtained as a preferable component to Al_2_O_3_ precipitation on it, and its morphology was investigated by scanning electron microscopy. The mechanical properties of the alloy after the oxygen blowing treatment are discussed in this work.

## 1. Introduction

From the mid-20th century, metals foundry has become increasingly attractive in light alloys fabrication—especially in Al casting alloys, as they are generally used in the automotive and aerospace industries. These alloys show advanced properties, such as low thermal and electrical resistivity, in addition to a relatively low density [1]. Unfortunately, the mechanical properties of aluminum casting alloys require improvement. Usually, strengthening of Al alloys is achieved by a traditional alloying process, by the addition of different compounds added to affect metal microstructure formation and consequently its mechanical properties [2,3,4,5,6,7]. Other works also exhibit the improvement of mechanical properties by applying the ultrasound method, which affects metal solidification [8,9,10].

One of the more suitable methods for this issue is obtaining reinforced composite alloys—especially in-situ composites formation by reactive gases; e.g., Wu and Reddy showed the reinforcement of Al-Si alloy by SiC produced by methane bubbling [11]. Zheng and co-authors showed the in-situ formation of AlN reinforcements by N_2_ bubbling [12], though it is important to note that the use of commercial nitrogen gas does not lead to the formation of reinforcements because of the hydrogen content in environmental moisture. Some works deal with the blowing treatment of oxygen-containing gases into Al alloys [13,14]. In these works, authors showed collapse of the oxygen bubbles, which leads to melt enrichment by the oxide skins and an increase of the alloys’ viscosity. They also found that gas bubbles collapse when the partial oxygen pressure reaches 21%. The formed oxide skins prevent the floating of the bubbles, affecting the porosity formation that is essential for the stable foams fabrication industry.

Aluminum alloy oxygen blowing treatment has been used occasionally. It was found that Al-Si melt was contaminated by hydrocarbons, which subsequently decomposed to their components—hydrogen and carbon. Because carbon is inert in the melt, hydrogen is responsible for the oxide bubble collapse. Therefore, one of the best solutions to the issue is to enrich the melt by titanium hydride, as was stated by Elliott [15]. Moreover, the technological approach of oxygen blowing of the preliminary saturated aluminum casting alloy by titanium hydride was described in detail in [16]. Authors demonstrated that the formation of aluminum oxides initiates the precipitation of refractory intermetallic compounds, and hydrogen serves as a moving asset due to its burning on the overheated melt surface.

Thus, two approaches of oxide bubbles collapse in Al alloy melt are known; namely, gas enrichment by maximum oxygen content of 21% [13,14] and a preliminary saturation of the melt with hydride-enriched compound [16]. In the current work, we propose an alternative approach of aluminum alloy melt overheating for gas bubbles collapse initiation. Additionally, the aim of the presented work is a study of the influence of the oxygen blowing process on the microstructural formation and mechanical properties in the overheated AlSi_7_Fe_1_ casting alloy. The formation of aluminum oxides during the process and their transfer into the gaseous phase will be described in the work. The understanding of this phenomenon can lead to the economically beneficial approach of melt refining from the oxide inclusions and even processing of aluminum scrub.

## 2. Materials and Methods

Commercial AlSi_7_Fe_1_ casting alloy was used as a bulk material. The composition of the alloy is given in Table 1.

Ingot of AlSi_7_Fe_1_ alloy was melted in a resistance furnace into a 750 mL corundum crucible. One gram of titanium hydride (TiH_2_) packed in Al foil was incorporated into the melt, followed by the oxygen blowing process through a quartz pipe with an inner diameter of 2 mm, as also described in [16].

**Figure 1:**
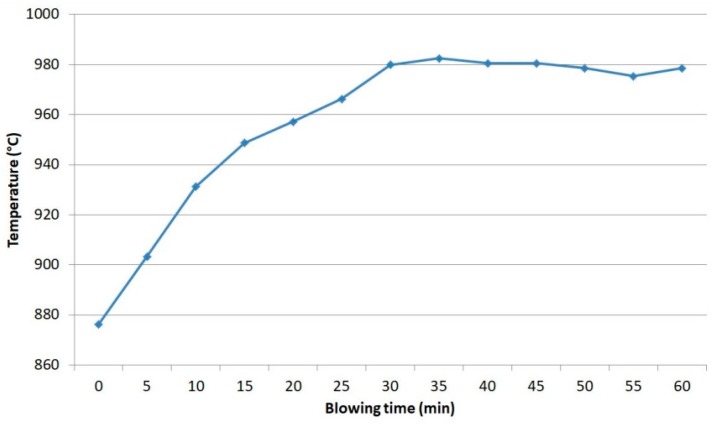
AlSi_7_Fe_1_ casting alloy temperature mode as a function of oxygen blowing.

The evaluation of the overheating temperature was done in a preliminary TiH_2_ saturated aluminum alloy by a K-type thermocouple (Chromel/Alumel). The obtained results are shown in Figure 1, and a steady-state experimental overheating temperature of 980 °С was set as the overheating temperature in the work.

A blowing treatment was made by a technical oxygen gas at the rate of 0.1 m^3^/h for 1 h. Then, the melt was cooled down to 650 °С—the temperature of the pouring process. The initial alloy was poured at the same temperature. The pouring process was made into a green sand mold and subjected to the followed investigations.

Microstructural studies were carried out with an Olympus BX53MRF-S optical microscope (Tokyo, Japan). The obtained specimens were examined after etching by Keller–Wilcox’s reagent (3 mL HCl, 5 mL HNO_3_, 1 mL HF, and 190 mL H_2_O). The average α-Al grains and the percentage of the eutectic phase area were measured by Clemex image analysis software (Longueuil, QC, Canada).

Electron microscopy images were taken by Tescan MIRA 3 FEG-SEM (Brno, Czech Republic) equipped with an energy dispersive spectroscopy (EDS) system by Oxford instruments with X-Max^N^ detector (Abingdon, UK).

The mechanical properties were measured by a testing machine Instron 3385 (Norwood, MA, USA) according to the ASTM E8M [17]. Each sample was subjected to 3 measurements and their average values are presented in results.

## 3. Results

**Figure 2:**
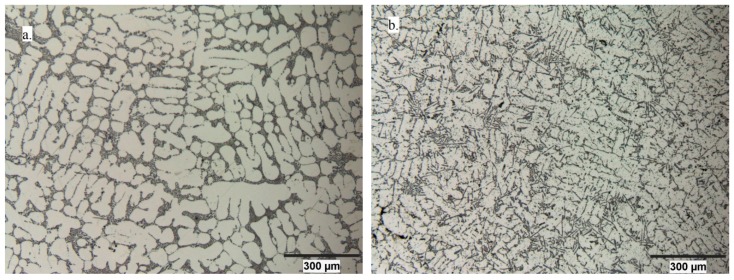
Optical microstructures of the (**a**) initial alloy and (**b**) alloy treated by oxygen blowing.

Microstructural evaluation of initial alloys and alloys subjected to oxygen blowing is shown in Figure 2. The structural characterization calculations were made based on these images—namely, the average α-Al grain length and the eutectic Si phase area composition, which are presented in Table 2.

As is evident from the obtained microstructures, the oxygen blowing treatment causes an average grain size reduction. The primary formed dendritic structure disappeared, and new finer α-Al grains with a homogeneously distributed Si network was formed. This statement is also supported by the structural characterization changes shown in Table 2.

**Figure 3:**
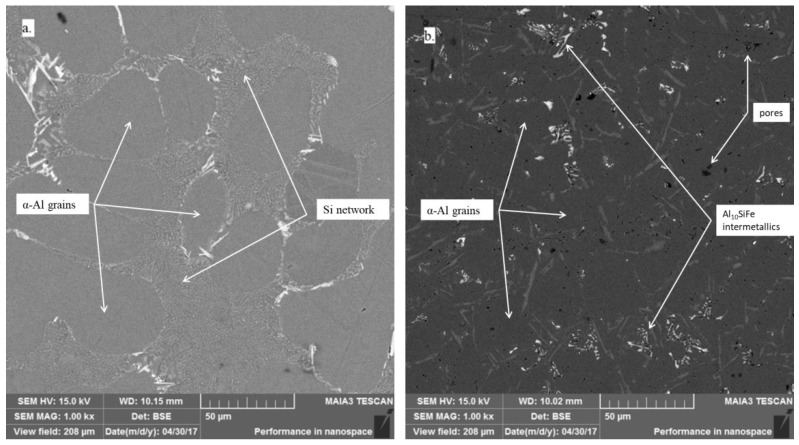
Scanning electron microscopy images of (**a**) the initial alloy and (**b**) the alloy treated by oxygen blowing.

Electron microscopy studies were applied to reveal any intermetallic compound formation during the oxide blowing treatment. This study allows an understanding of the formation of intermetallic compounds’ chemical and microstructural components, and further prediction of the alloys’ final properties. Scanning electron microscopy images are presented in Figure 3.

**Figure 4:**
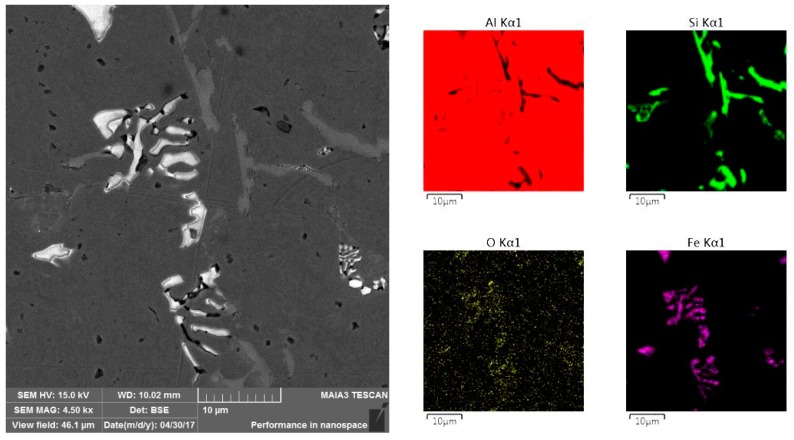
Scanning electron microscopy image of the Al_10_SiFe complex intermetallic compound and energy dispersive spectroscopy (EDS) mapping analysis of the image.

The initial alloy microstructure (Figure 3a) consists of α-Al grains (grey areas marked by arrows) with a Si network surrounding them (white network marked by arrows). However, the treated alloy microstructure (Figure 3b) consists of non-uniform α-Al grains (grey areas marked by arrows) and multi-component eutectics containing Si grains with a complex intermetallic Al_10_SiFe compound (white asymmetric compounds marked by arrows) which are enlarged and shown in Figure 4.

Figure 4 demonstrates the typical morphology of the complex intermetallic compound with a Chinese script-like shape. This intermetallic forms as a result of the appearance of aluminum oxide during the oxygen blowing process, and it attracts iron in the melt.

**Figure 5:**
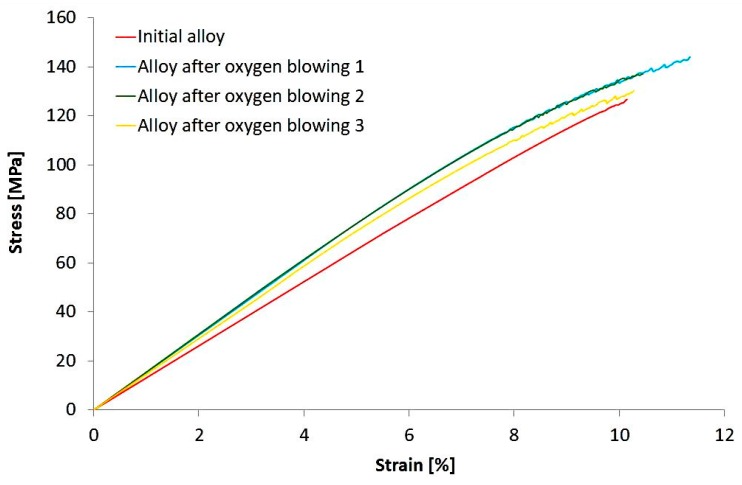
Stress-strain curves for initial alloys and alloys treated by oxygen blowing.

The obtained stress-strain curves are presented in Figure 5, and the mechanical properties are demonstrated in Table 3.

The mass balance of the initial alloy as well as the mass balance of the alloy subjected to the oxygen blowing treatment were calculated, and the results are presented in Table 4.

## 4. Discussion

The obtained AlSi_7_Fe_1_ alloy subjected to the oxygen blowing process contained a high concentration of micro-pores. Usually, porosity in aluminum alloys exhibits a round shape form, resulting as a decrease of the hydrogen solubility during metals solidification in a green sand mold. In the present work, an irregular shape of the pores was observed. Based on the presented results, it is obvious that oxygen blowing treatment is the main reason for their formation. We assumed that pores were formed into the melt by the oxygen bubble collapse mechanism, as also described by Babcsán and co-authors [18].

Generally, Al-Si alloys’ strength influenced by their chemical composition and the stability of the formed aluminum oxides. The melt temperature is one of the most important parameters affecting the alloys’ strength. Different volatile compounds can be formed in the Al-Al_2_O_3_ system, including Al, AlO, Al_2_O, and Al_2_O_2_. They were first investigated in the middle of the 20th century by Brewer and Searcy [19], and later by Hoch and Johnston [20], who showed the formation of the stable gaseous sub-oxide Al_2_O in the temperature range of 1050–1600 °С where the following chemical reaction takes place:

4Al(l) + Al_2_O_3_(s) ⟺ 3Al_2_O(g) (1)

However, Zhang et al. showed in their work that Al_2_O is a metastable compound in the temperature range of 900–1000 °С [21]. Consequently, the formation of this metastable oxide compound is the main reason for the strength reduction due to cracks and porosity healing inhibition.

It is obvious that the volatile sub-oxides form on the melt surface as well. This phenomenon is supported by the mass loss calculations in the process. Based on the obtained micro-porosity structure, we assumed that the majority of the bubbles collapsed on the melt surface, oxygen evaporated, and the remained oxide compounds (in the form of skins) reinforce the alloy. This assumption is confirmed by the formation of a fine aluminum microstructure. According to Stock’s equation, the observed pores formed as a result of a low floating rate of the bubbles. Even taking into account the minimum possible viscosity of the alloy (pure aluminum), it should take more than 24 h for the bubble floating into a crucible of 90 mm height. Moreover, micro-porosity negatively affects metals’ mechanical properties; Mirza and Chen proposed the estimation of the yield strength reduction with the appearance of porosity [22].

On one hand, the yield strength of the alloy subjected to the oxygen blowing decreased because of the eutectic phase compaction. On the other hand, the tensile strength of the treated alloy increased according to the reduction of the average grain size due to the Hall–Petch equation [23]:

σ = σ_0_ + K·d^−1/2^ (2)

where σ is the strength of the alloy, σ_0_ is a materials constant for the starting strength for dislocation movement, d is the average microstructure grain size, and K is the strengthening coefficient (specific to each material).

The main reason for the relatively low tensile strength simultaneously with the nearby values of the yield strength is the formation of the intermetallic compound Al_5_SiFe. This intermetallic compound forms as the result of the oxygen blowing treatment, which acts as a nucleant for its formation. This phenomenon has been confirmed by the work of Cho et al. [24], as well as by Borodianskiy and Zinigrad [25]. Moreover, based on Kato et al.’s report [26], intermetallic compounds interact with silicone matrix, reducing Si segregation to the grain boundary, resulting in the reduction of grain boundary brittlement and an increase in alloys’ ductility. Çetin and Kalkanli confirm in their work that Al_5_SiFe propagates the formation of porous microstructure, as mentioned above [27].

In the current work, we state that the modification of the alloy subjected to the oxygen blowing occurs by the heterogeneous crystallization mechanism by means of aluminum oxide nucleation, as also confirmed by [28]. In their work, Jackowski and co-authors described that Al_2_O_3_ is the most preferable nucleate because of its good wettability by the compounds presented in the Al-Si alloy. In the current work, Al_10_SiFe complex intermetallic compound was formed at the temperature range of 611–629 °С followed by the precipitation of the aluminum oxide on it. Furthermore, our statement was confirmed by a fine microstructure formation resulting from the melt overheating up to 980 °С, which is the initial temperature of the alloy heterogeneous crystallization [29].

## 5. Conclusions

In the presented work, the formation of aluminum oxide in AlSi_7_Fe_1_ casting alloy subjected to the oxygen blowing treatment was investigated. First, an Al_10_SiFe complex intermetallic compound was formed and it acted as a nucleant for the aluminum oxide’s appearance, and its Chinese script shape morphology was studied as well.

The influence of this component on the microstructure and mechanical properties of the alloy was investigated. Both the melt overheating up to 980 °С and a presence of aluminum oxides led to the refinement of α-Al grains, resulting in the enhancement of alloys’ tensile strength and ductility. Simultaneously, the alloys’ yield strength reduced due to the formation of micro-porosity in the metal.

## Figures and Tables

**Table 1:** Aluminum AlSi_7_Fe_1_ casting alloy chemical composition (wt %).

Si	Mg	Fe	Cu	Mn	Al
7.42	0.23	1.06	0.15	0.23	Balance

**Table 2:** Structural characterization of the initial and treated by oxygen blowing alloys.

Alloy	Length α-Al (µm)	Eutectic Phase Composition (%)
Initial alloy	50.56	24.80
Treated alloy	32.69	29.18

**Table 3:** Mechanical properties of initial alloys and alloys treated by oxygen blowing.

Mechanical Property	Initial Alloy	Treated Alloy
Tensile strength (MPa)	127	134
Yield strength (MPa)	120	96
Ductility (%)	10.1	10.7

**Table 4:** Mass balance calculations of initial alloys and alloys treated by oxygen blowing.

Calculated Parameter	Initial Casting Process	Oxygen Blowing Treatment Process
Mass change (%)	+0.12	−1.56
